# Influence of the Recent Earthquake Shocks in Charleston upon Health

**Published:** 1887-03

**Authors:** F. Peyre Porcher

**Affiliations:** Physician to the City Hospital, Charleston, S. C.


### INFLUENCE OF THE RECENT EARTHQUAKE SHOCKS IN CHARLESTON UPON HEALTH.

By F. PEYRE PORCHER, M. D.,
**Physician to the City Hospital, Charleston.**

In addition to the natural alarm and fright — especially among women and children — caused by the recent calamity, where so many persons were so suddenly brought face to face with death under the most appalling circumstances conceivable, and in every variety of situation and condition, I have gathered the following facts from personal observation and inquiry. They are interesting because rather unique in our experience in this country.

As the result of the shocks, some persons were instantly attacked with nausea accompanied by vomiting, which recurred or persisted in several cases for days.

Mrs. M. was nauseated during the first shock (Aug. 31) and had repeated attacks afterward, with vomiting and nervo-electrical disturbance. Miss M. was nauseated at the first vibratton, and before she could leave her chamber. Mrs. K. was absent Aug. 31, but in all the subsequent shocks she suffered from giddiness, with a feeling as if the floor was trembling under her feet, and experienced "instead of sadness, a tendency to laughter." There was no tingling or electrical disturbance. She was nauseated but once. Mr. S. and Mr. H. had nausea, and both had colic and diarrhœa following, on the night of August 31. Mrs. G. suffers from extreme nausea after every shock. She was sound asleep after the second shock (Friday, 28th Sept.), yet woke up nauseated. I could give numerous examples of a similar character.

Mrs. M., Mrs. S. and Mrs. D. experienced for a long time a feeling, whether real or imaginary, of tremors — as if the floor or the earth were incessantly moving under their feet. Similar sensations were felt by persons living in the country. Many of those residing on the coast declare that delicate and almost imperceptible vibrations are constantly felt. Mrs. C., living some distance from this city, experienced a sensation "as if she was suspended in mid air." One of my patients, Mrs. B., remarked: "The earthquake leaves you powerless; a flush and tingling come on before a shock — the whole body pulsating, with tears; everybody cried on account of pain in their knees and legs!" Mr. F. informs me that he would constantly anticipate the recurrence of shocks by his sensations — nervousness, tingling, etc. Two young gentlemen, on the islands eighty miles from Charleston, had their eyes suddenly filled with tears (Aug. 31), not to be repressed, but not caused by alarm, or fears for their personal safety, for the danger there was not imminent. These examples could be multiplied by the relation of others, if more extensive inquiries were made.

The commotion, attrition, and tremblings of vast masses of earth might not only squeeze out water, sand and gasses, but very naturally generate electricity on a tremendous scale, which would affect those who were specially sensitive.

I have the statements of many persons that they experienced decided electrical disturbances, which were repeated upon the successive recurrence of the shocks. These were generally tingling, pricking sensations, like "needles and pins," affecting the lower extremities. A few may be cited: Judge ——, in a town far removed from this city, immediately after the great shock, inquired of Mr. A., "How do you feel?" The reply was, "As if pricked by needles and pins." To which he rejoined, "That is precisely my experience." The Rev. ——, when asked as regards the effects produced upon him, stated that it was "an electrical thrill — after and with all the shocks; exactly what he had before experienced in his frequent use of the electric battery." His sister was similarly affected. Mr. S. feels the nervous sensation *before* the shocks occurring at night — never in the day.

A child of one of my patients would give immediate notice of the approoch of a shock, night or day, but oftener at night, by calling aloud; being far more keenly alive to them than the adult members of the family, or than his mother, who generally kept awake. The inability to move his leg, caused by a recent sprain of the knee from which he suffered, was greatly increased after each occurrence of shocks. His leg would then remain stiff and contracted. Another, Mr. B., assures me that he could always anticipate and know of the approach of a shock by his peculiar nervous electrical sensations. Miss. K. says she could feel the effects of the shocks "beginning in her big toe," and extending with a tingling sensation upward. One gentleman has been completely relieved of his rheumatism; another, who for months was nervous, depressed, and entirely unable to attend to any business, has regained his former activity and energy through the influences generated by the repeated convulsions of nature.

A gentleman on a visit to Columbia, awakening from a dream, experienced such sensations as to inquire whether a shock had not just occurred; his daughter replied affirmatively, and "that everything was electrified." In another individual (Dr. F. informs me) the vibrations affected his dreams, and upon awakening he would announce what had occurred. In a recent attendance upon Mrs. D., she stated that on three occasions *before* a shock occurred, she became dizzy and sick, and grasped a post or other convenient object for support.

With regard to my own experience: Whilst seated in the N. E. R. R. depot, awaiting the arrival of friends, the great earthquake disturbance occurred, when the foundations of the earth were felt to move and surge under our feet. This drove us out of the room and across the open street. I sought the protection and support of a telegraph pole on the opposite pavement in order to avoid the flying horses and vehicles, which could scarcely be seen through the sudden darkness — not wholly caused, I think, by the dust, which latter attracted the attention of every one. In this position I was not conscious of the second shock, except that I experienced suddenly great *nervous exhaustion* about the back and loins, and a sensation almost of pain. I am convinced, upon reflection, that it was wholly electrical, and similar to that experienced by others. I was not nervous, or particularly sensitive to the danger, as I wrote letters, and slept the remainder of the night, in a four-story brick dwelling which had lost every chimney, and was otherwise rent and injured; On another occasion, whilst engaged in adjusting a bandage for a fractured clavicle in a girl, aged thirteen, a pretty severe vibration occurred ; I remained (*impavidum ferient ruinæ*), but the patient fled.

The following occurred in the *News and Courier* of a recent date :

"The concurrent testimony of the most trustworthy experiences indicates that there is an electrical accompaniment to the earthquake, whether as cause or effect. A lady, while she was in the market, felt the same tingling nervous sensation which she experienced during the disturbance on the night of August 31st ; she was again seized with the same sensation, but in a more pronounced degree. The electricity appeared to permeate her whole body, which was affected just as it would have been by the continuous discharge through her system of the fluid from a galvanic battery."

I have seen in my own practice two cases of miscarriage and one of premature birth, and have heard of several others, consequent upon the earthquake — though no special inquiries have been made.

Several cases of mental disturbance, owing to anxiety and prolonged loss of rest, some of them persistent, have occurred in my own experience. One of these has not yet recovered her sanity. Exposure in tents and to the weather has been productive of much general ill consequences as was natural—namely: catarrhal fevers, bronchitis and an aggravation of lung diseases generally, and of those dependent on derangements of the nervous and uterine systems. Several persons have, by reason of the successive shocks, been reduced to a highly nervous condition, where their emotion could not be repressed. Dr. F., of Georgetown county, reports two such persons who will require to be sent to a more quiet abode. One of these occurred in an active, strong, hardworking man, who was not unnerved by the first commotion, but who had finally to succumb, with his nerves completely shattered. He confessed with pain and mortification the deplorable state to which he had been reduced. Hysterical attacks have also been developed in those previously well.

The most important premonitions of the great movement of the earth were three: 1. Almost entire absence of storm, thunder, lightning and other electrical disturbances during the entire summer, with a very prolonged drought, which still continues. 2. A peculiar appearance of the sky and the atmosphere on the evening of August 31, observed and commented on by several persons. Very bright orange sunsets still prevail (Nov. 18). 3. Very oppressive heat and sultriness experienced by every one just before 10 o'clock p. m., August 31. The smell of sulphur and gas was plainly observed after the convulsion of nature. This was also noticed by those on the incoming train of the N. E. Railroad, and was probably caused by the emanations from the extensive marshes of this region. With the exception of a little sulphur in the "green sand," that substance does not exist in the geological formation; one hundred to one thousand feet of marl underlie the superficial strata. Notwithstanding, a strong smell of sulphur is repeatedly observed before and after each shock by many residents of the coast near Georgetown, as I am credibly informed.

One lady of very delicate nostril — "*femina emunctœ naris*" — whom I attend in Summerville, where the premonitory rumbling and terraqueous disturbances are more marked, experiences a sensation of the smell of "sulphur and rhubarb" *before* each shock.

Dr. A. A. Moore, of Camden, S. C., in his report to the State Board of Health, September 23d, speaking of the effects of the earthquake shocks, says: "They at first naturally created much consternation among our population, and have undoubtedly had a very deleterious effect upon sick and feeble persons, being followed by much nervous prostration and other unpleasant symptoms. Even upon well and robust people their effects have been striking in some instances. Some have described their sensations as being similar to those experienced after a shock from an electric battery. Others have experienced a very marked feeling of debility in their lower extremities; others have had vertigo, nausea, etc. Some, again, who were not affected by these unpleasant symptoms in the beginning, are now troubled by them."

Dr. H. M. Stuart, of Beaufort, also reports that "it was a curious fact to note the number of persons, both male and female, particularly females, who were nauseated by the shock of the earthquake. This, I suppose, is attributable to fear, but these persons, although acknowledging themselves frightened at first, disclaimed any fear during the subsequent shocks, and continued to suffer from nausea. In a few other cases the effect of the nervous shock from the above cause was of a much more serious nature. In one case — that of a young lady — it caused hysterical convulsions."

It is several years since I read the *Kosmos*, but, if I remember rightly, Humboldt remarks therein on the "headache" and other morbid sensations which were experienced during the prevalence of earthquakes.

Domestic animals seemed to be exceedingly sensitive. Dogs howled, poultry were unusually noisy, and horses greatly alarmed. I give one instance which occurred to-day (December 2). A very intelligent dog belonging to Mr. W., walked into the breakfast room about 8 a. m., got up from the fire-place, before which he had stretched himself, went into a corner of the room, and remained there trembling and shivering. His master remarking that the animal was mad, his little son, who had a similar experience previously, replied, "No; it is an earthquake." The true explanation was in a few moments realized in a well-developed trembling.

The same dog in awakening his master, who was asleep in a hammock during the disturbance of August 31st, nearly tore off his clothes, so great was his excitement.—*Med. News.*

